# Functional or structural impairment of flow-mediated epicardial vasodilation may precede coronary microvascular dysfunction

**DOI:** 10.1016/j.ijcha.2025.101606

**Published:** 2025-01-09

**Authors:** Ines Valenta, Salwa Mikhail, Ashwin Singh Parihar, Sudhir Jain, Thomas H. Schindler

**Affiliations:** a Mallinckrodt Institute of Radiology, Division of Nuclear Medicine, Cardiovascular Medicine, Washington University School of Medicine, St. Louis, MO, USA; b Department of Medicine, Division of General Medicine, Washington University School of Medicine, St. Louis, MO, USA; c John T. Milliken Department of Internal Medicine, Cardiovascular Division, Washington University School of Medicine, St. Louis, MO, USA

**Keywords:** Coronary artery calcifications, Coronary microvascular dysfunction, Endothelial function, Myocardial blood flow, Positron emission tomography

## Abstract

**Background:**

The aim was to investigate whether functional and/or structural impairment of flow-mediated epicardial vasodilation (IEV) may precede coronary microvascular dysfunction (CMD) in a cardiometabolic risk population.

**Methods:**

^13^N-ammonia positron emission tomography/computed tomography evaluated global and longitudinal myocardial blood flow (MBF) during pharmacologically induced hyperemia and at rest. Normal coronary microvascular function (nCMF) was defined by a myocardial flow reserve (MFR = MBFstress/MBFrest) of ≥ 2.0, while an abnormal MFR of < 2.0 (predominantly due to decreases in hyperemic MBF) denoted classical CMD. Normal flow-mediated epicardial vasodilation (NEV) was defined as longitudinal hyperemic MBF gradient < -0.10 mL/g/min, whereas a value ≥ -0.10 mL/g/min signifiedIEV. Patients were grouped as follows: group 1 (G1): nCMF and NEV (n = 93); group 2 (G2): nCMF and IEV (n = 62), and group 3 (G3): CMD and IEV (n = 78). From non-gated CT, a semiquantitative four-point scoring system was used to indicate coronary artery calcifications score (CCS).

**Results:**

The prevalence of diffuse coronary artery calcification was highest in G1 with 51 %, followed by G3 with 46 % and G2 with 34 %. The extent of CCS was mild-to-moderate and did not differ significantly among groups (p = 0.222). Overall, IEV was present in 60 %, while there was a comparable prevalence of IEV between G2 and G3 (27 % and 33 %, p = 0.27). The hyperemic MBF gradient was highest in G2, intermediate in G3, and lowest in G1 (−0.22 ± 0.11 and −0.18 ± 0.10 vs. 0.03 ± 0.08 mL/g/min; p < 0.001, respectively).

**Conclusions:**

In this cardio-metabolic risk population, in about one third of these symptomatic patients functional and/or structural impairment of flow-mediated epicardial vasodilation may precede coronary microvascular dysfunction.

## Graphical abstract

**Figure:**
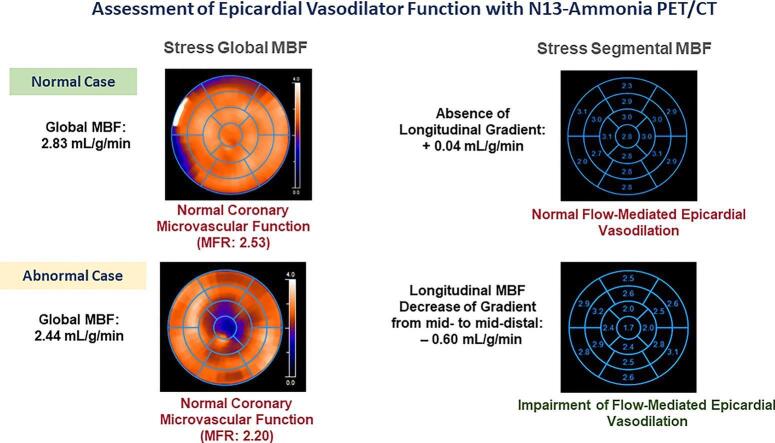

## 1 Introduction

The coronary endothelium is pivotal in the modulation of coronary circulatory function and in maintaining the structural and functional integrity of arterial wall [1], [2]. Apart from ascertaining a flow-mediated and thus endothelium-dependent epicardial vasodilation via the release of nitric oxide to the vascular smooth muscle cells, the endothelial-derived and nitric oxide exerts import anti-thrombotic and anti-atherosclerotic effects in the subintimal arterial space [1]. And indeed, numerous invasive clinical investigations to assess coronary artery function [3] have demonstrated an impairment of endothelium-dependent coronary vasomotor function as an independent predictor of the initiation and/or progression of CAD and future cardiovascular events. Invasive approaches for the assessment of epicardial endothelial function are time consuming and cumbersome as they necessitate quantitative coronary angiography to assess endothelium-dependent changes of the lumen of the epicardial artery in response to specifically acetylcholine-stimulated endothelial release of nitric oxide or flow-mediated and thus endothelium-dependent vasodilation during hyperemic coronary flow via intracoronary application of adenosine, or via sympathetic stress stimulation with cold pressor testing [1], [4], [5]. Conversely, invasive approaches for the assessment of endothelial dysfunction of the epicardial artery do not allow a general clinical application for the diagnosis of coronary arterial dysfunction, cardiovascular prognostication, and treatment monitoring thereof. Numerous PET flow studies [6], [7] have demonstrated that the non-invasive identification of reductions in myocardial flow reserve or coronary microvascular dysfunction (CMD) is predictive of subsequent cardiovascular events and clinical outcome in cardiovascular risk individuals. Conversely, PET computation and quantification of an abnormal hyperemic longitudinal MBF gradient from the base to the apex of the left ventricle during hyperemic flows, has been demonstrated to yield more specific information on diffuse CAD and/or impaired flow-mediated epicardial vasodilation in cardiovascular risk individuals [8], [9], [10]. The relationship between an impairment of flow-mediated epicardial vasodilation (IEV) and/or coronary microvascular dysfunction (CMD) remains to be further elucidated. In this respect, we aimed to evaluate whether an IEV may precede CMD, and its dependency on the hyperemic flow increases in a cardiometabolic risk population.

## 2 Methods

### 2.1 Patient population

**Fig. 1:**
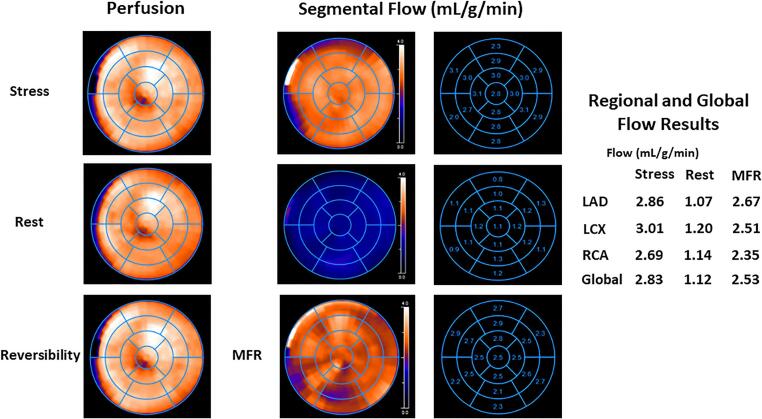
Normal myocardial perfusion and MBF study with ^13^ N-ammonia PET/CT in 70-year-old women with atypical chest discomfort and dyslipidemia. (a) Regadenoson-stress and rest ^13^ N-ammonia PET/CT images in corresponding short-axis (top), vertical long-axis (middle), and horizontal long-axis (bottom) slices. There is normal and homogeneous radiotracer uptake of the left ventricular wall on stress and rest ^13^ N-ammonia PET/CT to signify normal perfusion. (b) Polar map display with normal myocardial perfusion on PET/CT count images during stress, and rest widely ruling out hemodynamically significant obstructive CAD (right panel). Regional MBF quantification signifies normal hyperemic MBFs and MFR in all three major coronary artery territories of the LAD, LCx, and RCA with a global hyperemic MBFs of 2.83 mL/g/min, global rest MBF of 1.12 mL/g/min, and corresponding global MFR of 2.53 outlining normal coronary microvascular function (middle panel). Following, longitudinal MBFs in the mid and mid-to-distal myocardial segment of the LV corresponding to the vascular territories of the LAD (segments: 7–8 and 13–14), LCx (segments: 11–12 and 16), and RCA (segments: 9–10 and 15) were assessed using the 17-segment model [15]. In this case, LAD mid-segments 7 and 8 with stress MBF of 3.0 and 2.9 mL/g/min, but without a decrease in the mid—to-distal segment 13–14 with stress MBF of 3.1 and 3.0 mL/g/min demonstrating the absence of an abnormal longitudinal MBF gradient during hyperemic MBFs to signify normal flow-mediated epicardial vasodilation in the LAD distribution. This can also be seen for the LCx distribution, mid-segments (11 and 12) with stress MBF of 3.0 and 3.1 mL/g/min, respectively, and mid—to-distal segment (16) with stress MBF of 3.0 mL/g/min. Similarly for the RCA distribution, mid-segments (9 and 10) with stress MBF of 2.7 and 2.8 mL/g/min, respectively, and mid—to-distal segment (15) with stress MBF of 2.8 mL/g/min. When calculating the mean stress MBF values for the mid and mid-to-distal segments of the LAD, LCx, and RCA distribution, then there is virtually no decrease rather a mild increase in segmental MBF from the mid- (mean: 2.91 mL/g/min) to mid-to-distal segments (mean 2.95 mL/g/min) and, thus, no abnormal longitudinal MBF gradient during hyperemic MBFs is appreciated to denote normal flow-mediated epicardial vasodilation (lower-middle panel).

**Fig. 2:**
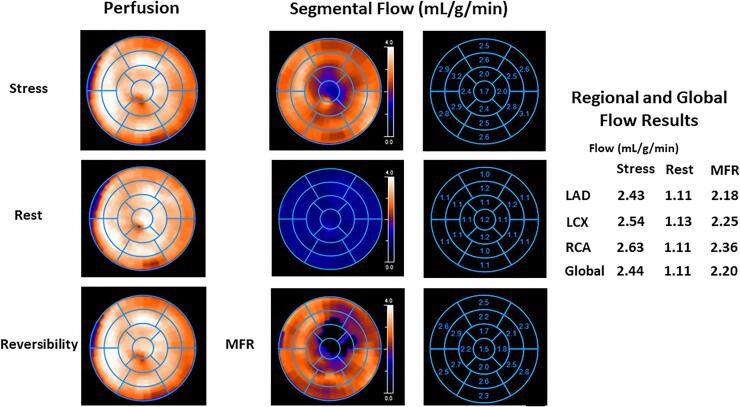
Normal myocardial perfusion and MBF study with ^13^ N-ammonia PET/CT in 60-year-old women with atypical chest, shortness of breath and medically treated arterial hypertension. (a) Regadenoson-stress and rest ^13^ N-ammonia PET/CT images in corresponding short-axis (top), vertical long-axis (middle), and horizontal long-axis (bottom) slices. There is normal and homogeneous radiotracer uptake of the left ventricular wall on stress and rest ^13^ N-ammonia PET/CT to signify normal perfusion. (b) Polar map display with normal myocardial perfusion on PET/CT images (right panel). Regional MBF quantification signifies normal hyperemic MBFs and MFR in all three major coronary artery territories of the LAD, LCx, and RCA resulting in a global hyperemic MBFs of 2.44 mL/g/min, global rest MBF of 1.11 mL/g/min, and corresponding global MFR of 2.20 compatible with normal coronary microvascular function (middle panel). Conversely, when evaluating the mean stress MBF values for the mid and mid-to-distal segments of the LAD, LCx, and RCA distribution, there is marked decrease in mean segmental MBF from the mid- (mean 2.80 mL/g/min) to mid-to- distal segments (2.20 mL/g/min) and, thus, an abnormal longitudinal MBF gradient during hyperemic MBFs to signify a marked impairment of flow-mediated epicardial vasodilation.

The initial study population consisted of 343 symptomatic patients having a low likelihood of flow-limiting obstructive CAD predominantly with medically treated cardiovascular risk factors such as dyslipidemia, arterial hypertension, and type 2 diabetes mellitus, who were consecutively referred for stress-rest myocardial perfusion and flow assessment with ^13^N-ammonia positron emission tomography/computed tomography (PET/CT) to evaluate the presence of focal and advanced obstructive CAD and/or CMD between 1^rst^ November 2018 and 31th December 2022. Only patients with normal pharmacologic stress and rest ^13^N-ammonia PET/CT myocardial perfusion images, that widely rule out significant downstream flow-limiting effects of focal CAD lesions or high-risk CAD [2], as well as normal wall motion analysis on gated ^13^N-ammonia PET, were included for study purpose [8], [11]. In this respect, patients with peak stress transient ischemic cavity dilation of the left ventricle during maximal vasomotor stress on gated PET images, as a reflection of stress-induced diffuse myocardial ischemia owing to hemodynamically obstructive left main and/or multivessel CAD, were excluded from the study [2]. Further, ^13^N-ammonia PET/CT concurrently determined global and longitudinal myocardial blood flow (MBF) during pharmacologically stimulated hyperemia and at rest. Normal coronary microvascular function (nCMF) was defined by a myocardial flow reserve (MFR = MBFstress / MBFrest) of ≥ 2.0, while an abnormal MFR of < 2.0 denoted CMD [7]. Abnormal reductions in MFR were then subgrouped into “classical” (predominantly related to decreases in hyperemic MBFs) and “endogen” (predominantly related to increases in resting MBF) CMD [7]. Furthermore, normal flow-mediated epicardial vasodilation (NEV) was defined as longitudinal hyperemic MBF gradient < -0.10 mL/g/min, whereas a value ≥ -0.10 mL/g/min signified IEV [8], [12]. Patients were grouped as follows: group 1 (G1): nCMF and NEV (Fig. 1); group 2 (G2): nCMF and IEV (Fig. 2), and group 3 (G3): CMD and IEV. Further, 110 patients with endogen CMD, as defined with a MFR < 2.0 due to increases in resting MBF (≥1.2 mL/g/min) and with NEV, were not included as they did not serve for study purpose [7] and as they were reported in a more recent publication [8]. Thus, the final study population consisted of 233 symptomatic patients as described in Table 1. It is important to note that 239 of the initial 343 study patients from the data base were included in previous investigations assessing and characterizing coronary vasomotor function in various risk populations in a different setting [8], [10], [11], [13]. Vasoactive medications such as calcium channel blockers, angiotensin-converting enzyme inhibitors, angiotensin II receptor blockers, statin as well as b-blockers, and diuretics were withheld at least 24 h before PET/CT myocardial perfusion- flow examination [11]. All study patients refrained from caffeine-containing beverages for at least 24 h and from smoking for at least 12 h prior to the cardiac PET/CT study. The study was approved by the Washington University in St. Louis (No.201812037), and each participant signed a clinical approved informed consent form [14].

**Table 1:** Patient characteristics among groups 1–3.

	**Group 1**	**Group 2**	**p Value**	**Group 3**	**p Value**
N (numbers)	93	62	*0.041*	78	0.348
Sex (female/male)	47/46	49/13	*0.0001*	58/20	0.001
BMI, kg/m^2^	37.4 ± 10.3	35.1 ± 8.77	0.064	36.7 ± 8.4	0.589
Age, yrs	57.9 ± 10.9	53.1 ± 12.3	*0.016*	60.3 ± 12.4	0.175
CCS	0.25 ± 0.29	0.17 ± 0.27	0.112	0.19 ± 0.26	0.211
Cardiovascular risk factors					
Hypertension, n	52 (55.9)	38 (61.3)	0.194	53 (67.9)	0.931
Dyslipidemia, n	53 (56.6)	28 (45.2)	*0.013*	51 (65.4)	0.863
Diabetes mellitus, n	25 (26.8)	12 (19.4)	*0.043*	18 (23.1)	0.314
Smoking, n	6 (6.5)	2 (3.2)	0.163	5 (6.4)	0.766
Family history of CAD, n	8 (8.6)	11 (17.7)	0.504	19 (24.4)	*0.041*
Obesity, n	60 (64.5)	36 (58.1)	*0.032*	62 (78.5)	0.876
Morbid obesity, n	33 (35.5)	12 (19.4)	*0.0033*	19 (24.4)	0.070
Lipid status					
Total cholesterol, mg/dl	163 ± 43	171 ± 47	0.367	164 ± 34	0.921
LDL cholesterol, mg/dl	92 ± 34	100 ± 38	0.255	85 ± 25	0.156
HDL cholesterol, mg/dl	52 ± 17	50 ± 14	0.542	55 ± 14	0.308
Triglyceride, mg/dl	126 ± 62	135 ± 62	0.473	126 ± 85	0.997
Glucose, mg/dl	114 ± 35	106 ± 27	0.164	115 ± 35	0.788
hs CRP, mg/l	3.81 ± 2.59	6.11 ± 7.23	0.117	11.41 ± 16.90	*0.017*
HbA_1c_, %	6.01 ± 1.13	5.94 ± 1.02	0.478	6.29 ± 1.09	0.397

Values are mean ± standard deviation (SD). Numbers (%). P values versus group 1 (G1) (Mann-Whitney *U* test for independent samples).

BMI = body mass index; CCS = coronary artery calcium score; LDL = low-density lipoprotein; HDL = high-density lipoprotein;

hsCRP = high-sensitivity C-reactive protein; HbA1c = glycosylated hemoglobin.

### 2.2 Myocardial perfusion and flow studies with PET/CT

^13^N-ammonia PET determined myocardial perfusion and myocardial blood flow (MBF) in mL/min/ with serial image acquisition (Biograph mCT PET-CT scanner, Siemens Healthineers, Erlangen, Germany) as described previously [8].After conducting the topogram to define the axial field of view and a low-dose CT scan (120 kV, 30 mA) for attenuation correction, ^13^N-ammonia PET determined ed myocardial perfusion and MBF in ml/min/g with serially acquired images and a two-compartment tracer kinetic model during regadenoson-stimulated hyperemia and at rest, respectively [8]. PET image acquisition was conducted during regadenoson-stimulated hyperemia (0.4 mg intravenous bolus injection over 10 and 20 s interval) and commenced immediately after injection of ≈370 MBq ^13^N-ammonia as a bolus followed subsequent infusion of 10 ml saline solution over 30 s (0.33 ml/sec) via infusion pump (e.g. Aitecs Syringe Pump), and also at least 40 min later at rest, for a total duration of 10-min list mode PET acquisition, respectively. Subsequently, static images of myocardial perfusion during pharmacologic-induced hyperemia and at rest were analyzed visually on reoriented short-and long-axis myocardial slices and semi quantitatively on the corresponding polar map from the last static 10-min transaxial PET image. Semiquantitative evaluation of ^13^N-ammonia PET perfusion images were conducted with a standard 17-segment model and a five-point grading system by two expert observers [15]. Summed stress score (SSS), summed rest score (SRS), and summed difference score (SDS) were determined. An SSS < 4 was deemed normal, 4–8 mildly abnormal, 9–13 moderately abnormal, and > 13 severely abnormal perfusion defect. Further, a SDS ≥ 2 signified a reversible perfusion defect, whereas < 2 was deemed as normal. In this direction, the extent of regional reversible perfusion defects on ^13^N-ammonia PET images was scored according to the SDS value. An SDS of 2–4, >5–8, and > 8 defined mild, moderate, and severe reversible perfusion defects, respectively. Only patients with a SDS < 2, and normal wall motion on gated PET were included in for study analysis [8], [15]. Image interpretation was visually performed in consensus by two nuclear cardiologists. In the case of disagreement between observers, consensus was obtained in a joint reading.

As regards the assessment of MBF in ml/g/min, left ventricular (LV) contours and input function region were acquired automatically with minimal operator intervention using Corridor4DM software (Cardiac; 4DM PET CFR) version 2016 (INVIA Medical Imaging Solutions, Ann Arbor, MI) [8]. Starting with intravenous ^13^N-ammonia i.v. injection, serial transaxial emission images were acquired (dynamic frames: 1 × 5 sec, 12 × 10 sec, 4 × 30 sec, 4 × 60 sec, 1 × 120 sec) with PET/CT, and time-activity curves from the first 2 min in concert with a two-compartment tracer kinetic model were applied to calculate regional and global MBF in ml/g/min [8], [15].On the polar map of the last 600-s image set, regions of interest (ROIs) were aligned to the territories of the main three coronary arteries using the 17-segment model [15]. Using the Corridor4DM software package, MBF was determined in ml/g/min. Regional MBFs of the LAD, LCx, and RCA territory were averaged on a polar map and the calculated mean MBF of the left ventricle was defined as global MBF. Subsequently, longitudinal flows, MBFs in the mid and mid-distal myocardial segment of the LV corresponding to the vascular territories of the LAD (segments: 7–8 and 13–14), LCx (segments: 11–12 and 16), and RCA (segments: 9–10 and 15) were assessed [15]. Basal segments (LAD: 1–2, LCx: 5–6, and RCA: 3–4) and the apical segment (LAD: 17), however, were not included for this evaluation due to possible count variability caused by the membranous septum, by a certain variability in locating the last apical slice, and by partial volume errors resulting from object size at the apex [15], [16]. Heart rate, blood pressure, and a 12-lead electrocardiogram were acquired continuously during each MBF assessment. From the average of heart rate and systolic blood pressure during the first 2 min of each image acquisition during maximal hyperemia and at rest, the rate-pressure product (RPP) was derived as an index of myocardial workload [11]. A decrease in MBF from mid to mid-distal LV myocardium (ml/g/min) was defined as longitudinal, base-to-apex MBF gradient. Alterations in the longitudinal, base-to-apex MBF gradient from regadenoson induced hyperemia stimulation to rest were defined as stress-to-rest alteration in longitudinal, base-to- apex MBF gradient (Δ longitudinal MBF gradient = longitudinal MBF gradient during hyperemia minus longitudinal MBF gradient at rest). To account for inter-individual variations in coronary driving pressure, an index of coronary vascular resistance (CVR) was determined as the ratio of mean arterial blood pressure (mmHg) to MBF (ml/g/min). As resting MBF is dependent on the myocardial workload, it was normalized to the RPP (averaged during the first 2 min of image acquisition; MBF divided by RPP multiplied by 10,000). This again served to calculate the corrected MFR (Hyperemic MBF during regadenoson divided by NMBF at rest). In addition, a visual four-point scoring system was used to indicate coronary artery calcifications (0 = normal, 1 = mild, 2 = moderate, and 3 = severe calcifications) of the LAD, LCx, and RCA on the non-gated and low-dose CT for attenuation correction [17]**.**

### 2.3 Statistical analysis

Data are presented as the mean ± SD for quantitative and absolute frequencies for qualitative variables. For comparison of differences, appropriate t-tests for independent samples were used. A comparison of MBFs and MFR among the different groups was performed by 1-way analysis of variance (ANOVA) followed by Scheffe multiple comparison test. Since CCSs followed a skewed distribution, the CCSs were also logarithmically transformed (log-CCS). Multivariate analysis was performed by means of a linear regression model adjusting for the following a priori selected predictors of the hyperemic longitudinal MBF gradient: CCS, MFR, gender, age, BMI, SBP, total cholesterol, LDL cholesterol, HDL cholesterol, triglycerides, glucose, high-sensitive C-reactive protein, and glycosylated hemoglobin. Differences in prevalence of gender, cardiovascular risk factors and coronary microvascular (dys)function among groups were analyzed with the x^2^ test. All test procedures were 2-tailed, and *p* ≤ 05 was considered statistically significant. All statistical analyses were performed with SPSS for Windows 27.0 (SPSS).

## 3 Results

### 3.1 Clinical and study characteristics

The clinical characteristics of the cardio-metabolic risk study population are displayed in Table 1. The prevalence of diffuse coronary artery calcification was 45 % (104/233), while it was highest in G1 with 51 % (47/93), followed by G3 with 46 % (36/78) and G2 with 34 % (21/62). Conversely, the mild-to-moderate extent of the CCS was comparable among groups (Table 1).

### 3.2 Prevalence of IEV, hemodynamic findings, and MBF parameters

After excluding 110 patients with endogen CMD in the initial study population, IEV was present in 60 % (140/233) in G2 and G3. Further, G1 with normal MFR and NEV was more prevalent than IEV without CMD in G2 (40 % vs 27 %, p = 0.041) and non-significantly higher than IEV with CMD in G3 respectively (40 % vs 33 %, p = 0.349), while the prevalence did not differ between G2 and G3 (p = 0.27).

At rest, heart rate and systolic blood pressure and corresponding RPP were higher in G2 than in G1, whereas they were the highest in G3 (Table 2). The latter was paralleled by a progressive increase in global resting MBF and global normalized MBF from G1 to G2 and G3. During regadenoson-stimulated hyperemic flow increases, heart rates had increased significantly from rest, while SBP had decreased significantly. Conversely, stress SBP were highest in G3, followed by G2, and lowest in G1, so that there was a progressive increase in stress RPP from G1 to G2 and G3, respectively. Global hyperemic MBF and global MFR tended to be higher in G2 than in G1, while lowest in G3, respectively (Table 2, Fig. 3a). The group comparison of global hyperemic MBF and global MFR in G2 and G3 was significant compared to G1, respectively (p < 0.0001 both by ANOVA). When the global hyperemic MBFs were related to the mean arterial blood pressure to account for possible interindividual variations in coronary driving pressure, the resulting measures of global CVR (mean arterial blood pressure/global MBF) widely mirrored the global MBF values at rest and during pharmacologic vasodilation for G1-3 studied (Table 2). Consequently, differences in coronary driving pressure during pharmacologically stimulated hyperemic coronary flows can be widely excluded as cause for alterations in global hyperemic MBF responses. Also here, the group comparison of global hyperemic CVR in G2 and G3 was significant compared to G1 (p < 0.0001 both by ANOVA).

**Table 2:** MBF and hemodynamic findings during PET/CT exam.

	**Group 1**	**Group 2**	**p Value**	**Group 3**	**p Value**
Flow Parameters					
MBF-Rest	0.97 ± 0.22	1.03 ± 0.20	0.110	1.30 ± 0.23	*0.0001*
NMBF-Rest	1.10 ± 0.30	1.11 ± 0.25	0.687	1.31 ± 0.27	*0.0001*
MBF-Stress	2.28 ± 0.49	2.51 ± 0.56	*0.009*	2.04 ± 0.39	*0.001*
MFR	2.37 ± 0.32	2.45 ± 0.44	0.228	1.58 ± 0.23	*0.0001*
Corrected MFR	2.16 ± 0.53	2.30 ± 0.53	0.114	1.60 ± 0.33	*0.0001*
CVR-Rest	102 ± 26	95 ± 19	0.115	78 ± 15	*0.0001*
CVR-Stress	38 ± 9	35 ± 7	*0.036*	41 ± 8	*0.003*
MBF Gradient-Rest	0.01 ± 0.05	−0.04 ± 0.05	*0.0001*	−0.03 ± 0.07	*0.0001*
MBF Gradient-Stress	0.03 ± 0.08	−0.22 ± 0.11	*0.0001*	−0.18 ± 0.10	*0.0001*
ΔMBF Gradient	0.03 ± 0.09	−0.18 ± 0.12	*0.0001*	−0.15 ± 0.11	*0.0001*
CVR Gradient-Rest	−0.89 ± 4.95	3.11 ± 4.19	*0.0001*	1.73 ± 3.72	*0.0001*
CVR Gradient-Stress	−0.47 ± 1.24	2.71 ± 1.51	*0.0001*	3.30 ± 1.98	*0.0001*
ΔCVR Gradient	0.41 ± 4.79	−0.40 ± 4.22	0.282	1.56 ± 3.72	0.085
Hemodynamics					
Rest HR, bpm	69 ± 11	71 ± 9	0.619	73 ± 10	*0.032*
Stress HR, bpm	91 ± 14	98 ± 15	*0.002*	96 ± 13	*0.029*
Rest-SBP, mmHg	131 ± 18	134 ± 16	0.409	139 ± 16	*0.010*
Stress-SBP, mmHg	117 ± 13	119 ± 14	0.352	121 ± 14	*0.044*
Rest-RPP	9195 ± 2147	9437 ± 1678	0.435	10114 ± 1705	*0.002*
Stress-RPP	10750 ± 2116	11857 ± 2459	*0.003*	11699 ± 2113	*0.004*
ΔRPP, pharmacologic – rest	1554 ± 1417	2420 ± 1752	*0.002*	1585 ± 2110	0.910

Values are mean ± standard deviation (SD). P values versus Group1 (Mann-Whitney *U* test for independent samples).

CVR = coronary vascular resistance; HR = heart rate; MBF = myocardial blood flow; MFR = myocardial flow reserve.

PET = positron emission tomography; RPP = rate-pressure product; SBP = systolic blood pressure.

**Fig. 3a:**
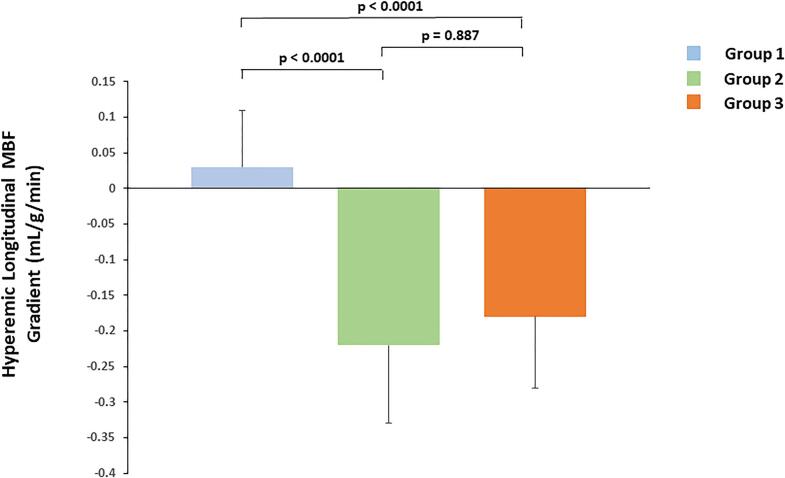
Global myocardial flow reserve (MFR) among groups. Global MFR for group 1, group 2, and group 3.

### 3.3 Longitudinal MBF gradient

There was virtually no rest longitudinal MBF gradient in G1, while a minor and comparable abnormal longitudinal MBF gradients were observed in G2 and G3, respectively (Table 2). During pharmacologically induced hyperemia stimulation, the observed hyperemic longitudinal MBF gradient was highest in G2, intermediate in G3, and lowest or none in G3, while it did not differ significantly between G2 and G3 (Fig. 3b). Similarly, the change in longitudinal MBF gradient from rest to hyperemia defined as Δ longitudinal MBF gradient (longitudinal MBF gradient during hyperemia minus longitudinal MBF gradient at rest) was significantly higher in G2 and G3 when compared to G1, while comparable between G2 and G3 (Table 2). The group comparison of the hyperemic longitudinal MBF gradient as well as of the Δ longitudinal MBF gradient differences in G2 and G3 was significant compared G1, respectively (p < 0.0001 both by ANOVA). To examine a possible relationship between the longitudinal MBF gradient and coronary microvascular function, the hyperemic MBF gradient was compared to the corresponding MFR. As displayed in Fig. 4a, Fig. 4b, the hyperemic MBF gradient as well as the Δ longitudinal MBF gradient correlated significantly with the MFR, respectively (r = 0.18, SEE = 0.51, p = 0.005 and r = 0.13, SEE = 0.50, p = 0.049), signifying the increase in coronary blood flow as a determinant of the longitudinal MBF gradient during hyperemic flows. These observations were further emphasized when G2 and G3 were analyzed separately from G1. Also here, the hyperemic MBF gradient and Δ longitudinal MBF gradient were significantly associated with the MFR, respectively (r = 0.20, SEE = 0.11, p = 0.019 and r = 0.21, SEE = 0.10, p = 0.012). Further, predictors of the hyperemic longitudinal MBF gradient were determined. In the study population (Table 3), on univariate analysis, CCS, MFR, sex, and BMI were significantly associated with the hyperemic longitudinal MBF gradient. In addition, we conducted a multivariate analysis to identify independent predictors of the observed hyperemic longitudinal MBF gradient, by which MFR, sex, BMI, age and SBP were identified as direct factors affecting the manifestation of the longitudinal MBF gradient during hyperemia stimulation.

**Fig. 3b:**
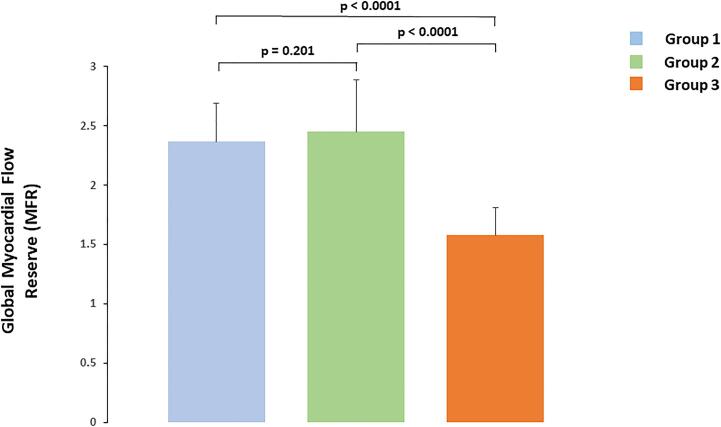
Longitudinal myocardial blood flow (MBF) gradient during hyperemic flow stimulation for the three study groups.

**Fig. 4a:**
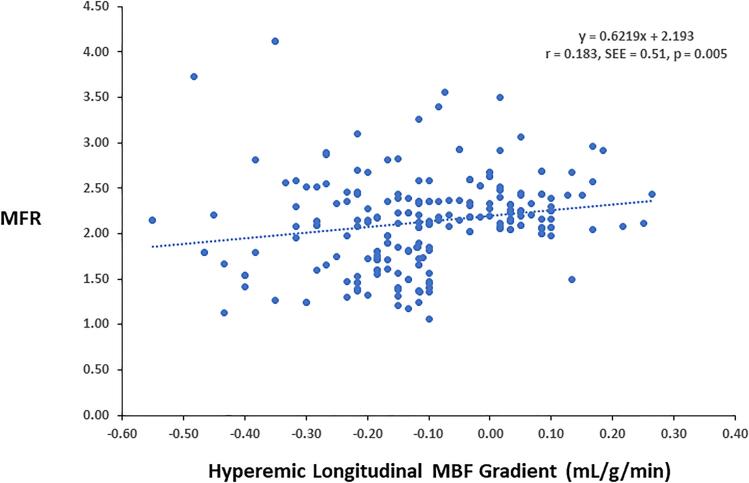
Correlation between hyperemic longitudinal MBF gradient and MFR in the entire study population. In this respect, normal flow-mediated epicardial vasodilation (NEV) was defined as longitudinal hyperemic MBF gradient < -0.10 mL/g/min, whereas a value ≥ -0.10 mL/g/min signified IEV.

**Fig. 4b:**
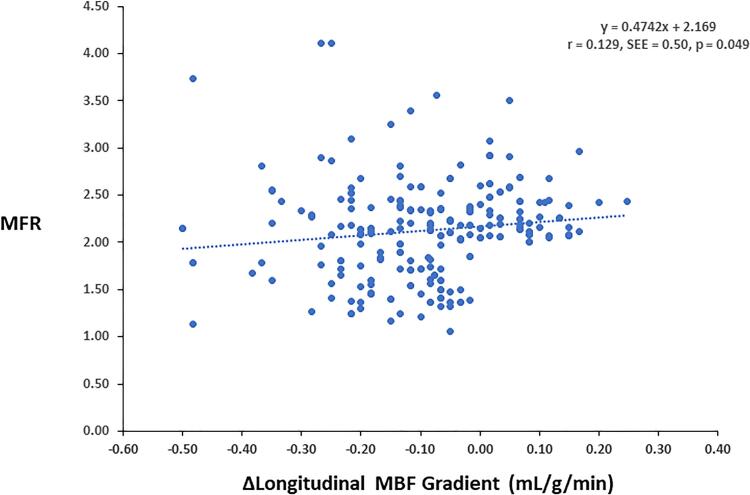
Correlation between longitudinal Δ MBF gradient and corresponding MFR in the entire study group. MBF = myocardial blood flow; SEE = standard error of the estimate.

**Table 3:** Predictors of impaired, flow-mediated epicardial vasodilation.

	**Hyperemic longitudinal MBF gradient**		
	**Univariate**	**Multivariate analysis**	
	**p Value**	**Standardized Coefficient**	**p Value**
CCS	0.026*	/	/
MFR	0.005*	0.215	0.002*
Gender	0.0001*	0.184	0.006*
Age,yrs	0.054*	0.174	0.032*
BMI, kg/m2	0.015*	0.207	0.002*
SBP at rest, mmHg	0.165	−0.133	0.044*
Total cholesterol, mg/dl	0.566	/	/
LDL cholesterol, mg/dl	0.870	/	/
HDL cholesterol, mg/dl	0.372	/	/
Triglyceride, mg/dl	0.885	/	/
Glucose, mg/dl	0.064	/	/
Hs CRP, mg/l	0.079	/	/
HbA_1c_, %	0.237	/	/

*Significant difference by analysis of variance.

Abbreviations as in Table 1.

## 4 Discussion

The current observations are unique as it demonstrates that PET-determined and non-invasive identification of structural and/or functional impairment flow-mediated epicardial vasodilation may indeed precede coronary microvascular dysfunction in about one-third of symptomatic patients in a cardiometabolic risk population. Notably, as an impairment in flow-related and, thus, endothelium-mediated epicardial vasomotor function is commonly seen as functional precursor of the CAD process, current observations may add a new dimension to the non-invasive assessment of the coronary flow regulation and cardiovascular prognostication.

Vascular smooth muscle–relaxing substances such as regadenoson decrease the resistance to flow at the site of the coronary arteriolar resistance vessels leading to a maximal or submaximal hyperemic MBF increase. Since regadenoson causes hyperemic MBF increases through vascular smooth muscle cell relaxation, the resulting hyperemic MBF increase is commonly referred to as a measure of a predominantly endothelium-independent flow response [2]. Conversely, shear-sensitive components of the coronary endothelium contribute by about 20 % to 25 % through flow-mediated coronary vasodilation to the overall hyperemic MBF increases during pharmacologic vasodilation [3], [5]. Conversely, diffuse CAD and/or functional impairment of the pre-described flow-mediated epicardial vasodilation during pharmacologically induced hyperemic flow increases may indeed cause down-stream fluid dynamic effects resulting in an abnormal longitudinal hyperemic MBF decrease in cardiovascular risk individuals. Structural and/or functional impairment of flow-mediated epicardial vasodilation in early stages of development of CAD is frequently paralleled by an impairment of flow-mediated epicardial vasodilation [3]. Under such conditions, the resistance to higher coronary flow increases and it may cause a progressive proximal-to-distal decrease in intracoronary pressure along the epicardial artery [5], leading to a longitudinal and gradual, base-to-apex decline in hyperemic MBF. The identification of an abnormal longitudinal hyperemic MBF gradient by means of PET could indeed reflect a promising and non-invasive probe of early structural or functional alterations of the CAD process predominantly at the site of the epicardial artery.

In the whole study population, IEV was present in 60 % of patients, while the prevalence of IEV did not differ significantly among those with normal or abnormal MFR. Thus, IEV may be present in about 27 % of patients despite normal coronary microvascular function that otherwise would be missed by conventional myocardial perfusion imaging or global assessment of the MFR. Furthermore, 33 % of patients had both IEV and CMD suggesting a progression of IEV to alterations of coronary vasomotor function also to the level of coronary arteriolar vessels. Such observations may be intriguing as they challenge the widely perceived contention that early alterations of coronary circulatory function start at the level of the coronary microcirculation favoring subsequent abnormalities in epicardial vasomotor function as functional precursor of CAD [6]. Interestingly, the severity of IEV, reflected by the extent of the hyperemic longitudinal MBF gradient, was significantly more pronounced in patients with normal hyperemic flow increases than in those with predominant classical CMD. This may be surprising at first sight but may be reconciled with the Hagen-Poiseuille law [5], [15], [16]**.** The latter describes that the resistance to flow is stringent on the length of the tube, the flow velocity and, notably, inversely on the fourth power of the vessel diameter. In the current study population, the extent of the hyperemic longitudinal MBF gradient weakly but significantly correlated with the degree of global hyperemic MBF increases, so that higher coronary flow increases were associated to some extent with a more pronounced hyperemic longitudinal MBF gradient, reflecting at least in part downstream effects of IEV on flow, and likely endothelial dysfunction of the epicardial artery. A mild sympathetic coronary vasoconstriction during hyperemic flow stimulation in the presence of epicardial endothelial dysfunction, inhibiting epicardial vasodilation, however, is also likely to have contributed to the hyperemic longitudinal MBF gradient [9].

It is important to keep in mind that early structural alterations of the CAD process or vessel stiffness may also hamper an appropriate flow-mediated epicardial vasodilation during hyperemic flow increases leading to an abnormal longitudinal perfusion or MBF gradient [9], [18], [19]. The prevalence of diffuse coronary artery calcifications, as a surrogate for coronary artery plaque burden, in the current study population was about 45 %. While the CT-determined coronary artery calcium score of mild-to-moderate severity did not differ significantly among groups, the prevalence was highest in the group with normal coronary circulatory function with 47 %, while 34 % and 46 % in those individuals with IEV and without or with CMD, respectively. Consequently, apart from IEV, non-obstructive diffuse CAD may also account for the observed hyperemic longitudinal MBF gradient in this cardiometabolic cardiovascular risk population. This also agrees with current observations of an association between CT-determined extent of coronary artery calcifications and the hyperemic longitudinal MBF gradient. Overall, in nearly half of this patient population, both structural and functional alterations of the epicardial artery likely have contributed to the manifestation of the longitudinal MBF gradient during higher coronary flows. Conversely, in those patients without evidence of coronary artery calcifications, the observed IEV can be related predominantly to an impairment of coronary endothelial function.

Notably, despite a comparable cardiometabolic risk profile and coronary artery calcification burden among the study groups, 55 % of these patients did present both NEV and nCMF, and thus had normal coronary vascular function at the level of both the epicardial artery and coronary arteriolar vessels, respectively. The reason for this intriguing observation remains uncertain but may be related, at least in part, to the recently described functional “obesity paradox” putting forth less vulnerability of epicardial endothelial function and the coronary arteriolar vessels in individuals with advancing obesity likely related to more favorable cardiometabolic profile, adipocytokine, and yet unknown factors [8]. It is also possible that in these patients with normal coronary vascular function, the duration of exposure to the cardiometabolic risk profile was not sufficiently long enough to effectively cause adverse effects on coronary circulatory function, less susceptibility of the arterial wall for adverse effects of the components of the metabolic syndrome, and/or yet unknown factors. Another consideration is that the hyperemic coronary flow increase may have been relatively low or not sufficiently high enough leading to a measurable abnormal longitudinal MBF gradient, despite the presence of mild-to-moderate epicardial artery stiffness.

On multivariate analysis, we observed BMI, sex, age, resting SBP, and MFR as independent predictors for the hyperemic longitudinal MBF gradient in the current study population. Since increases in BMI associates with components of the metabolic syndrome (low HDL cholesterol, insulin resistance, arterial hypertension, and CRP plasma levels), the independent predictive value of BMI for the longitudinal MBF gradient also signifies a complex interplay of low HDL cholesterol, insulin resistance, and systemic inflammation likely accounting to the manifestation of IEV with increases in body weight [8], [20], [21].

As it appears, gender differences may also have exerted some influence on the reported IEV in a study population that predominantly consisted of female patients. Given the age of the study population, most female patients can be assumed postmenopausal. A postmenopausal state is commonly accompanied by higher total cholesterol, LDL cholesterol, triglycerides, lower HDL levels, increases in body weight and arterial hypertension that favors the increased clinical manifestation of cardiovascular disease [22]. In this respect, it has been demonstrated that the postmenopausal state in women without hormone replacement therapy is indeed accompanied by coronary endothelial dysfunction, while long-term treatment with estrogen preserved endothelium-dependent coronary vasomotion [23]. Current observations therefore may agree with previous PET myocardial flow investigations in postmenopausal women [23], [24] but extend the latter findings, at least in part, to an IEV in a middle aged women population. MFR levels also proved to be an independent predictor of the abnormal hyperemic longitudinal MBF gradient. This may not be surprising as the resistance to flow is not only dependent on the vessel diameter but also related the flow velocity according to the Hagen-Poiseuille law [5], [25]. The independent predictive value of the MFR for the observed hyperemic longitudinal MBF gradient therefore outlines that a higher MFR leads indeed to a more severe longitudinal MBF gradient in the presence of IEV, that also agrees with prior non-invasive and invasive investigations in this field [16], [25].

### 4.1 Limitations

There are pivotal limitations worthy of considerations in interpreting these data. First, the study analysis was conducted in a cardiometabolic risk population. Thus, the results may not necessarily be translated to another patient population that differs in its cardiovascular risk profile. Second, we only performed a non-gated CT for attenuation correction of PET emission data instead of a dedicated and gated CT for an accurate detection and quantification of the coronary artery calcium score. Thus, we may have missed some mild coronary artery calcifications, and the reported coronary calcification burden likely may have been underestimated to a certain extent [26]. Third, since no contrast CT coronary angiography or invasive intravascular coronary ultrasound was performed in this population, early and subclinical CAD likely has remained undetected that may have contributed to the observed IEV in those individuals without coronary artery calcifications [27]. Fourth, the hyperemic longitudinal MBF gradient was quantified from the mid and mid-to-distal left ventricular segments along a relatively short longitudinal distance aiming to circumvent confounding count variability in the basal segments and partial volume effects in the apical segment on flow measurements as reported for our MBF quantification approach [8], [28] The quantified hyperemic longitudinal MBF gradient in the current study therefore is likely underestimated as reported by others [16] and as compared to mapping MBFs on a pixel basis that affords to measure the longitudinal flow gradient over a longer distance from the basal to apical segments [9], [19], [29]. Fifth, we did not include the endogen type of CMD with NEV that was predominantly observed in morbidly obese patients with a distinct different metabolic profile compared to obese individuals [8], favoring rather left ventricular hypertrophy and potential heart failure but not necessarily CAD as also evidence by the absence of an abnormal longitudinal hyperemic MBF gradient or NEV and widely absence of coronary artery calcifications. Further, global hyperemic MBF and MFR values were higher in G2 than in G1, while the cardiovascular risk profile did not differ among these groups (Table 1). When looking at hemodynamics or the rate-pressure product (RPP = heart rate x systolic blood pressure) during pharmacologic stress, the RPP during vasomotor stress was indeed mildly but significantly higher in G2 than in G1 that is also reflected by corresponding values of the coronary vascular resistance (CVR) (Table2). Thus, mildly higher coronary driving pressure during vasomotor stress may have indeed contributed to higher hyperemic stress MBFs and thus MFR in G2 compared to G1. Finally, given that the study is based on cross-sectional data, longitudinal follow- up investigations with PET/determined coronary morphology and flow are warranted in establishing a temporal relationship between observed structural and/or functional IEV and CMD.

## 5 Conclusions

In this cardio-metabolic risk population, in about one third of these symptomatic patients functional and/or structural impairment flow-mediated epicardial vasodilation may precede coronary microvascular dysfunction., that may add a new dimension in the non-invasive assessment of coronary flow regulation and cardiovascular prognostication, deserving further clinical investigations.

## CRediT authorship contribution statement

**Ines Valenta:** Writing – review & editing, Visualization, Software, Methodology, Formal analysis, Data curation, Conceptualization. **Salwa Mikhail:** Writing – review & editing, Visualization, Investigation, Formal analysis, Data curation, Conceptualization. **Ashwin Singh Parihar:** Visualization, Investigation, Formal analysis, Data curation, Conceptualization. **Sudhir Jain:** Visualization, Investigation, Formal analysis, Conceptualization. **Thomas H. Schindler:** Conceptualization, Methodology, Software, Validation, Formal analysis.

## Funding

This work was supported by a departmental fund from Washington University (No.12–3271-93128), St. Louis. Dr Schindler has received research grant support from GE Healthcare and NIH/NHLBI (1R01HL142297-01A1).

## Declaration of competing interest

The authors declare that they have no known competing financial interests or personal relationships that could have appeared to influence the work reported in this paper.

## Data Availability

Data will be made available on request.
